# Comparison of the Thrombogenicity of a Bare and Antithrombogenic Coated Flow Diverter in an In Vitro Flow Model

**DOI:** 10.1007/s00270-019-02307-5

**Published:** 2019-08-13

**Authors:** T. Lenz-Habijan, M. Brodde, B. E. Kehrel, C. Bannewitz, K. Gromann, P. Bhogal, M. Aguilar Perez, H. Monstadt, H. Henkes

**Affiliations:** 1 phenox GmbH, Lise-Meitner-Allee 31, 44801 Bochum, Germany; 2 OxProtect GmbH, Münster, Germany; 3 Department of Interventional Neuroradiology, The Royal London Hospital, London, UK; 4 Neuroradiological Clinic, Klinikum Stuttgart, Stuttgart, Germany

**Keywords:** Antithrombogenic, Flow diverter, Coating, Chandler loop, HPC, p48

## Abstract

**Background:**

Dual antiplatelet therapy is a pre-requisite for flow diverter (FD) implantation. The purpose of this study was to assess the thrombogenicity of the p48 FD, coated with the newly developed phenox Hydrophilic Polymer Coating (p48_HPC, phenox GmbH, Germany) in comparison with uncoated p48 FDs in an in vitro flow model (Chandler Loop).

**Methods:**

p48 and p48_HPC FDs were implanted into silicon tubes filled with whole human blood and incubated at 37 °C under pulsating flow. After 120 min, platelet count was determined in the blood. Platelet activation markers (PAR1) and formation of microparticles were analyzed in a flow cytometer. Fluorescence microscopy of CD42a positive cells and scanning electron microscopy was used to detect adherent platelets on the wire surface.

**Results:**

Platelets in contact with the uncoated p48 FDs are significantly more activated than those incubated with p48_HPC (73 ± 9% vs. 65 ± 6%, *p* < 0.05) and release more microparticles (1.8 ± 0.5 vs. 1.4 ± 0.4, *p* < 0.05). The platelet count after 120-min circulation in the Chandler Loop was significantly lower for the uncoated p48 compared to the p48_HPC indicating significantly greater adherence of the platelets to the p48 (71 ± 8% vs. 87 ± 5%, *p* < 0.05). SEM and fluorescent antibody imaging revealed minimal platelet adherence to the surface of the p48_HPC compared to the uncoated p48.

**Conclusion:**

The pHPC coating significantly reduces thrombogenicity of the p48 FD. This may help to reduce the risk of thromboembolic complications when using these devices. A reduction in antiplatelet therapy may be possible.

## Introduction

Flow diverters are low-porosity braided stents. They were designed to treat intracranial aneurysms without the need for coiling. They function by significantly altering the in-flow of blood into the aneurysm which promotes thrombosis, and simultaneously the struts of these stents act as a substrate for the formation of neo-endothelium. The end result is the reconstruction of the parent vessel and exclusion of the aneurysm [1]. Although there was initial concern regarding preservation of flow in side branches covered by these devices, the risk appears to be relatively minimal [2]. The pivotal Pipeline for Uncoilable or Failed Aneurysms (PUFS) trial, published in 2013 [3], showed a 6-month aneurysm exclusion rate of 73.6% that rises to 95.2% (*n* = 60/63) at 5 years [4].

Although flow diverter stents have revolutionized the endovascular therapy of aneurysms, one issue remains unsolved. Flow diverters have a high metal surface area and are therefore extremely thrombogenic. Inadequate platelet inhibition can have severe and life-threatening consequences on patients (e.g., transitory ischemic attack and ischemic stroke). It is therefore essential that under regular conditions (planned interventions), patients are commenced on dual antiplatelet therapy (DAPT) prior to the implantation of these devices and up to one year afterward. Nevertheless, in emergency procedures thrombocytes are acutely blocked using other drugs. This strategy minimizes the risk of thrombotic complications but consequently increases the risk of hemorrhagic complications throughout the body [5]. Additionally, the response to platelet inhibitors varies widely among patients, ranging from resistance to antiplatelet medications and consequently a higher risk of thrombogenic events, to hyper-responders that may have elevated rates of hemorrhagic complications [6–9]. Pharmacologic interactions between antiaggregants with widely used drugs (e.g., ibuprofen, omeprazole, simvastatin) may have fatal consequences for patients taking antiplatelet medications after stent implantation [10]. Patient incompliance with the antiplatelet medications is another known reason for thromboembolic complications [11]. A potential solution for this dilemma is the application of an antithrombogenic coating to the surface of the flow diverter or stent. Currently, there are two surface-modified flow diverters that have obtained CE mark—the Pipeline Shield (Medtronic, Irvine, California, USA) and the p48_HPC (phenox, Bochum, Germany). The Pipeline Shield device has a 3-nm phosphorylcholine coating covalently bound to the braid wires. Phosphorylcholine has been shown to reduce platelet adhesion and activation [12]. The p48_HPC has a newly developed glycan-based multilayer hydrophilic polymer coating.

The phenox Hydrophilic Polymeric Coating, pHPC, has recently been shown to reduce thrombogenicity when applied to nitinol surfaces [13]. Regardless of wetting and the biomaterial used, the initial adhesion of platelets appears to be mediated by GPIIb/IIIa binding to surface-adsorbed fibrinogen. Thereafter, various activation cascades lead to a conformational change in the GPIIb/IIIa receptor, increasing the affinity of the receptor for the von Willebrand factor (VWF) and fibrinogen [14]. This initial platelet adhesion is inhibited by the pHPC technology. The purpose of this study is to compare the thrombogenic profiles of the uncoated p48 (phenox, Bochum, Germany) flow diverter with the coated p48_HPC flow diverter using the in vitro Chandler Loop model. The Chandler Loop system is designed to simulate continuous human blood flow and has previously been used as an in vitro thrombogenicity testing method. It enables hemocompatibility testing of stents according to ISO 10993-4 [15].

## Materials and Methods

### Study Design

In this study, the thrombogenicity of the new polymer-coated p48_HPC flow diverter was compared to that of the uncoated p48 device in an in vitro flow model (Chandler Loop). In each individual experiment, three loops (one loop with a p48, one loop with a p48_HPC and one loop without implant as negative control) were run simultaneously. Thrombocyte adhesion and activation were assessed in five individual experiments after 2 h. Therefore, on the one hand thrombocyte activation was determined using a fluorescent-activated cell sorter (PAR1 and platelet-derived microparticles). On the other hand, cell adhesion was analyzed as cell loss in the blood after 2 h incubation using a cell counter and microscopically after CD42a immunohistochemistry under a fluorescence microscope and in the SEM.

### Specimens and Coating

The p48 flow diverter, constructed from 48 braided NiTi wires, was used as test device. The “phenox Hydrophilic Polymer Coating” (pHPC) was applied to the surface of the p48 stents. This coating is a newly developed glycan-based multilayer polymer. Two neurovascular devices coated with pHPC have been granted CE mark approval, the p48_HPC and the pCONUS_HPC.

### Blood Donation

Five healthy donors aged between 25 and 42 volunteered to have venous blood samples collected. The following parameters were analyzed for each blood donor via hematology analyzer (KX-21N, Sysmex, Norderstedt, Germany): total red blood cells, total white blood cells, hemoglobin, hematocrit, mean corpuscular hemoglobin (MCH), mean corpuscular hemoglobin concentration (MCHC), mean corpuscular volume (MCV) and total platelets. Standard values depend individually on the age and gender of the blood donor. They are provided by the company Sysmex on a device-specific basis. Exclusion criteria for participation in the study were an abnormal blood count measured via hematology analyzer and/or the use of drugs that act on either blood coagulation or platelet function, e.g., aspirin, clopidogrel, warfarin.

The number of platelets in the blood sample was recorded for each donor (not shown).

### Chandler Loop

**Fig. 1:**
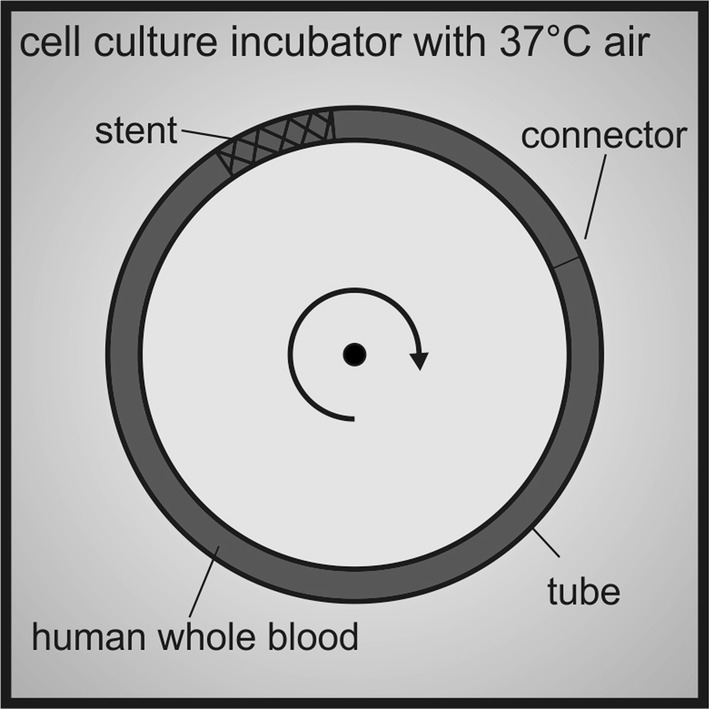
Schematic representation of the experimental setup (Chandler Loop model). The devices are incubated in rotating tubes in a cell culture incubator at 37 °C

The thrombogenicity of the p48 and p48_HPC was assessed using a Chandler Loop as schematically shown in Fig. 1. FDs were implanted in silicon tubes with an inner diameter of 3 mm. After stent deployment, the tubes were rinsed with 0.9% NaCl solution, then filled with heparinized (5 U/ml) whole blood from each of the volunteers. Introduction of the blood into the silicone tube was performed carefully to ensure air bubbles were not introduced. The tubes were sealed in an end-to-end fashion and incubated at 37 °C in air. Pulsatile flow was mimicked by means of discontinuous rotation of the blood in the tube. The rotation speed of the loops varies between 100 and 300 revolutions per minute, which simulates the pulse in the vascular system. This was continued for 120 min.

### Cell Count

After 120 min, the platelet count in the blood was determined using a cell counter (KX-21N, Sysmex, Norderstedt, Germany). A lower cell count, relative to the control, signifies thrombocytes that remain within the Chandler Loop or are adherent to the implanted stents; therefore, a higher thrombocyte cell count at 120 min represents reduced stent thrombogenicity.

### Fluorescence-Activated Cell Sorter (FACS)

After 120 min, the level of platelet activation in the blood was assessed. When blood comes in contact with foreign surfaces, platelet adhesion and activation take place. One way to measure thrombocyte activation is the cleavage of the platelet thrombin receptor PAR1. PAR1 belongs to the protease-activated receptor family. The proteolytic cleavage of the receptor by thrombin leads to activation and induces a number of reactions in the thrombocytes, including increased adhesion. Cleavage of PAR 1 occurs when blood contacts foreign bodies. The antibody used to assess PAR 1 binds only to uncleaved thrombin receptors; therefore, a reduction in the antibody binding indicates cell activation. Another marker of thrombocyte activation is the formation of microparticles. Platelet-derived microparticles (PMPs) are small extracellular vesicles, and these play a role in blood clotting and thrombus formation [16]. Both PAR1 and PMPs were analyzed. Whole blood was centrifuged, and the platelet-rich plasma was separated. Platelets were incubated with 50 U/ml hirudin, washed and incubated with a fluorescent-labeled antibody against the PAR1 receptor. After 60 min of incubation, antibody binding to the platelet surfaced was measured in a flow cytometer.

### CD42a-PE Immunohistochemistry

After incubation with the blood, the flow diverters were removed from the Chandler Loop and rinsed in phosphate-buffered saline (PBS) twice in order to remove any non-adherent cells. The adherent cells were fixated for 30 min with 0.4% paraformaldehyde in PBS, rinsed twice in PBS and then incubated with anti CD42a-PE antibody, diluted 1:10 in PBS. CD42a or platelet glycoprotein IX is a small membrane glycoprotein found on the surface of human platelets. After 60 min incubation in the dark, NiTi specimens were again rinsed in PBS twice, and analyzed using a fluorescence microscope (Olympus BX40, Olympus; Hamburg, Germany) equipped with a digital camera (Moticam 5, Motic, Wetzlar, Germany).

### Scanning Electron Microscopy (SEM)

SEM was performed using a LEO 1530 Gemini system (Carl Zeiss AG, Jena, Germany). For SEM analyses, specimens were incubated in whole blood for 120 min, rinsed in PBS twice, fixed with 3.7% glutaraldehyde (Sigma-Aldrich, Taufkirchen, Germany) in PBS for 15 min, dehydrated using an ascending series of alcohols, dried for 24 h at room temperature and sputtered with gold (Edwards Sputter Coater S150B9, Edwards Limited, Crawley, UK). SEM analyses were performed at the Ruhr University Bochum, Faculty of Mechanical Engineering, Department of Materials.

### Statistical Analysis

Statistical analysis was performed using GraphPad Prism Software (Version 3.03; GraphPad Software Inc., La Jolla, CA). After normal distribution was confirmed using the Kolmogorov–Smirnov test, differences between the p48_HPC and p48 were investigated using the paired student’s *t*-test. *p* values of less than 0.05 were considered statistically significant. The data represent the results from five individual experiments.

## Results

### The pHPC Coating Significantly Reduces Platelet Activation

**Fig. 2:**
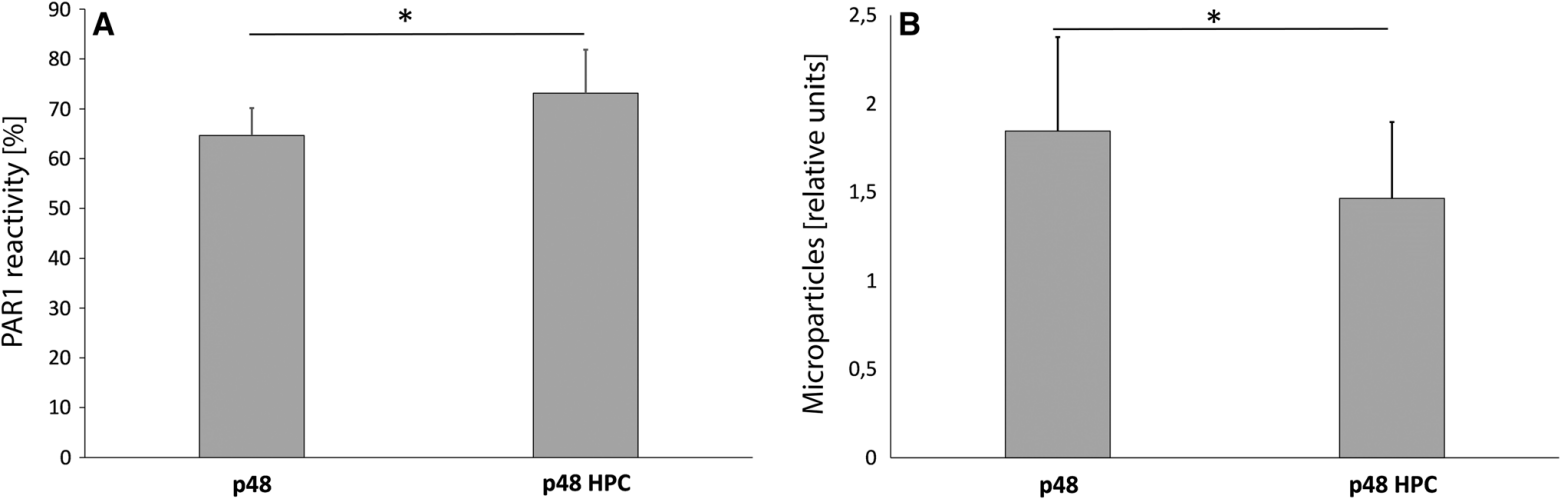
Thrombocyte activation after 120 min circulation in the Chandler Loop. The cleavage of the thrombin receptor PAR1 is used as an activation marker (**A**). In **B,** platelet microparticle release is used as a marker of platelet activation. PAR1 reactivity and microparticle release is shown relative to the control (blood directly after blood sampling). Thrombocyte activation is reduced significantly by the pHPC coating (Mean ± SD, asterisks denote significance at *p* ≤ 0.05; *n* = 5; *t*-test)

As markers of thrombocyte activation, both the PAR1 reactivity and the PMPs were assessed. The PAR1 reactivity is shown relative to the control (PAR1 blood levels measured directly after blood sampling = 100%; Fig. 2A). As shown in Fig. 2A, contact of the blood with foreign surfaces induces PAR1 cleavage, the PAR1 reactivity is decreased, compared to the freshly drawn blood, to 65 ± 6% (p48) and 73 ± 9% (p48_HPC; *p* < 0.05). The antibody binds only to uncleaved thrombin receptors, and therefore, a reduction in the antibody binding means an activation. Platelets in contact with the uncoated p48 for 120 min show a significantly decreased PAR1 reactivity compared to thrombocytes incubated together with the p48_HPC.

As shown in Fig. 2B, platelets in contact with the uncoated p48 form significantly more microparticles (factor 1.8 ± 0.5) than platelets incubated together with the pHPC-coated p48 (factor 1.4 ± 0.4).

### The pHPC Coating Significantly Reduces Platelet Adhesion

**Fig. 3:**
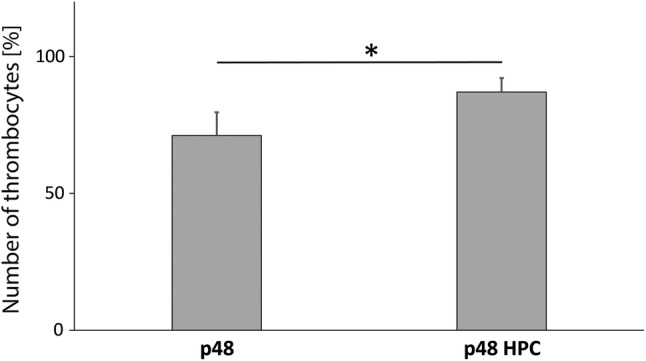
The platelet count after 120-min circulation in the Chandler Loop in percent of control. The “missing” thrombocytes remain in the tube or on the stent. The platelet count of the uncoated p48 is significantly reduced compared to the pHPC-coated p48 (mean ± SD, asterisks denote significance at *p* ≤ 0.05; *n* = 5; *t*-test)

**Fig. 4:**
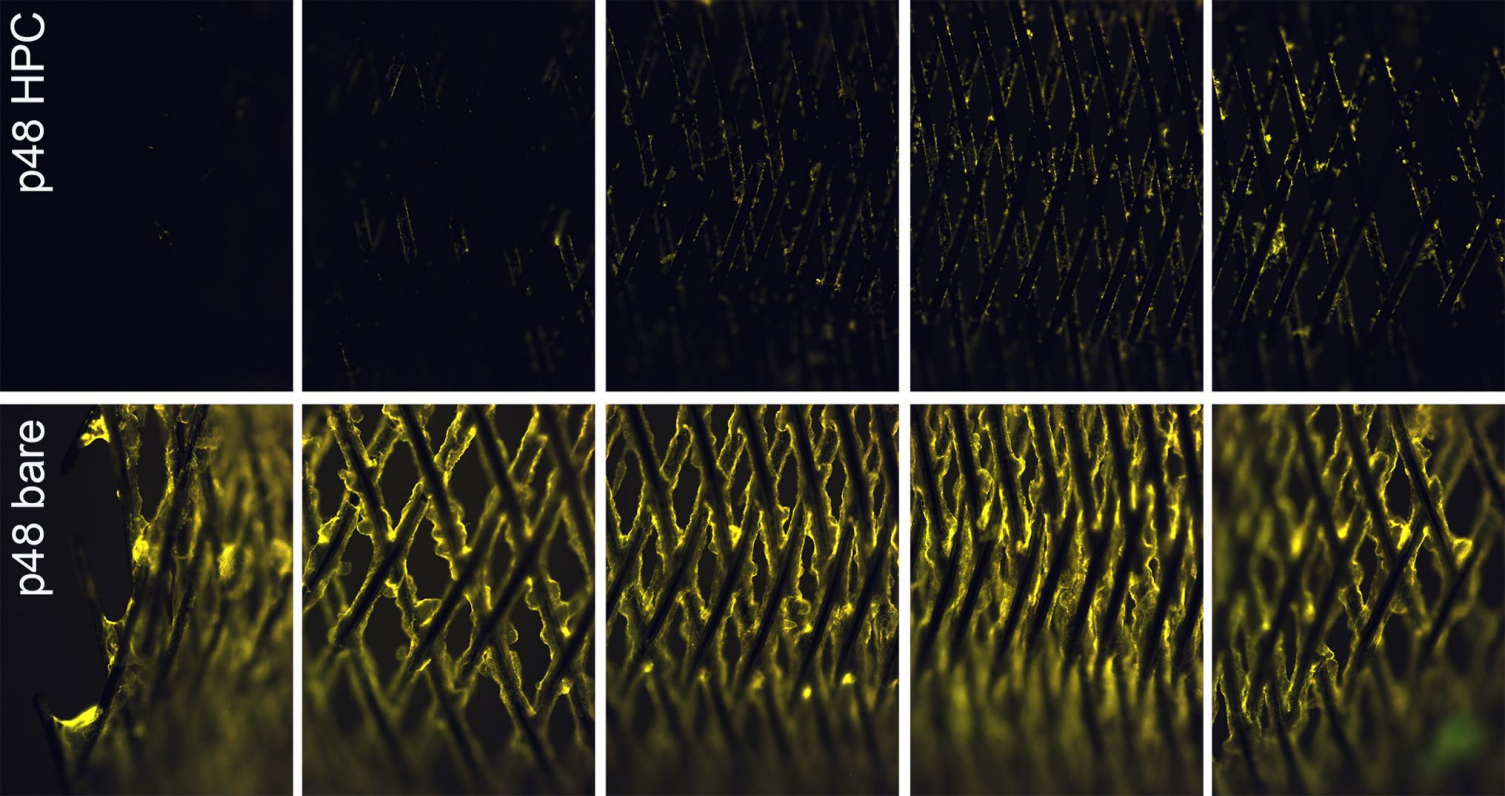
Representative fluorescence micrographs of uncoated (bare) and pHPC-coated p48 flow diverter stents. The specimens were incubated in whole blood for 120 min in the Chandler Loop under dynamic conditions. Adherent platelets were stained with a CD42a antibody (yellow fluorescence). While the coating almost completely prevents the adhesion of cells on the stent surface (upper row), the uncoated stent has a thick layer of platelets encapsulated inside a fibrin clot (lower row)

The platelet count after 120 min circulation in the Chandler Loop is shown relative to the control (blood directly after blood sampling = 100%; Fig. 3). The “missing” thrombocytes remain in the tube or are adherent on the stent. The platelet count of the uncoated p48 (71 ± 8%) is significantly reduced compared to the p48_HPC (87 ± 5%; *p* ≤ 0.05). Fluorescent microscopic pictures are shown in Fig. 4. The pictures in each row give an overview over the complete stents, p48_HPC in the upper row and p48 in the lower row. The uncoated p48 stent is covered by a thick layer of adherent fluorescent cells embedded in a thick fibrin clot, while on the p48_HPC fluorescence is much lower. Minimal fluorescent CD42a^+^ thrombocytes are detected on the p48_HPC with no clot formation visible under the fluorescence microscopy.

#### Scanning Electron Microscopy

**Fig. 5:**
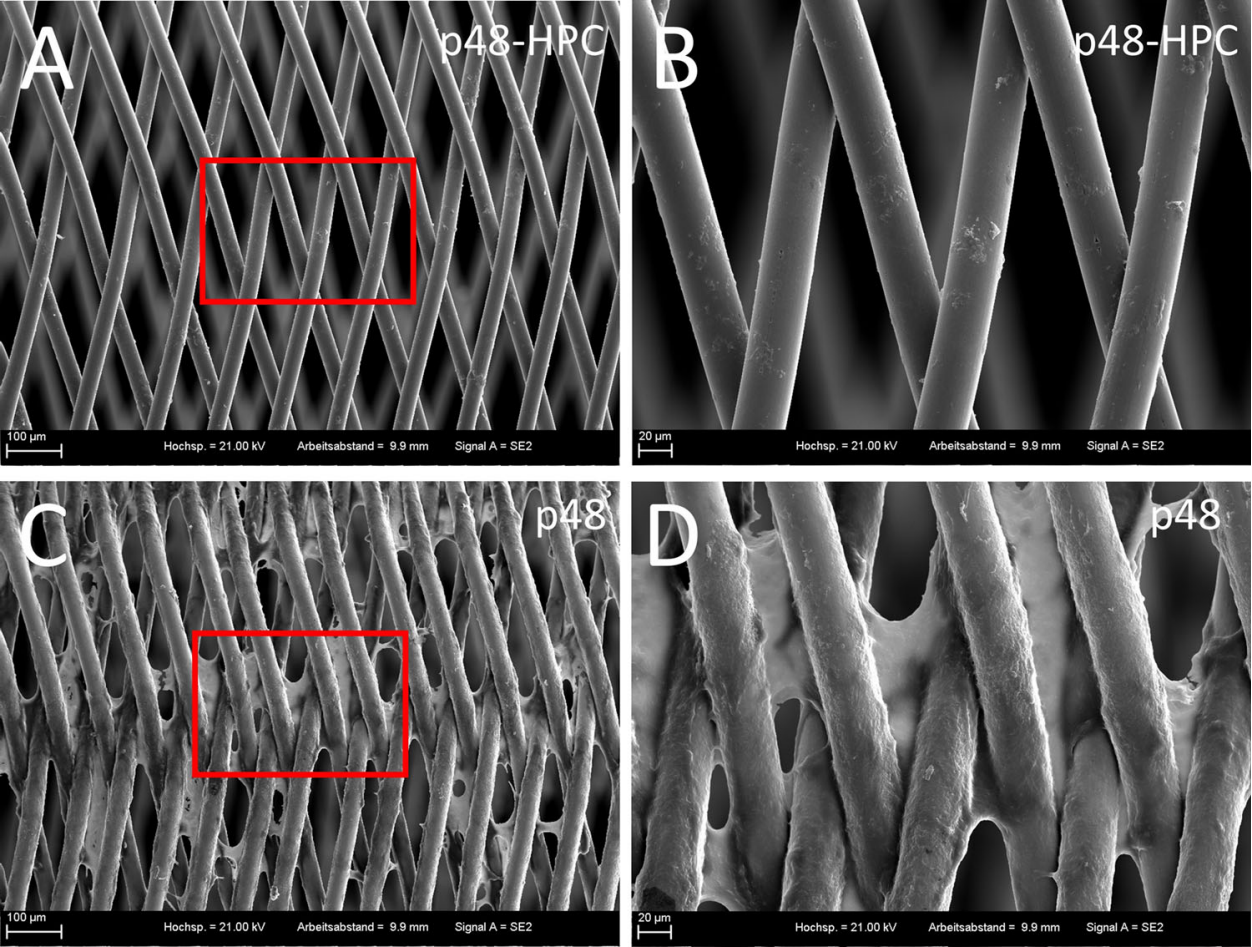
SEM micrographs of pHPC-coated (A + B) and uncoated (C + D) p48 flow diverter stents. The specimens were incubated in whole blood for 120 min in the Chandler Loop under dynamic conditions. Stents and adherent cells were fixated and sputtered with gold. The rectangle indicates the area shown under a higher magnification. While the coating almost completely prevents the adhesion of cells on the stent surface (A + B), the uncoated stent shows clot formation on and between the struts (C + D)

Scanning electron microscopy was used to assess the presence of fibrin clots and location of the adherent platelets on the wires of the stents. The braided wires of the coated p48_HPC stent show only a few cells sparsely distributed along their surface (Fig. 5A). Under higher-power magnification, minor defects in the surface of the wire surface are visible (Fig. 5B). There is no evidence of adherent clot on the p48_HPC after incubation with whole blood in the Chandler Loop. In contrast, there is dense clot seen on the surface of the wires of the uncoated p48 stent with dense clots seen at the crossing points of the braided wires (Fig. 5C). Under higher-power magnification, it is evident that the surface of the wire cannot be seen as it is completely covered in adherent clot (Fig. 5D).

## Discussion

In this study, the thrombogenicity of the new polymer-coated p48_HPC flow diverter was compared to that of the uncoated p48 device using a Chandler Loop model and various markers of thrombocyte activation. We have shown that there is a significant reduction in the surface thrombogenicity of the p48_HPC compared to the p48 as assessed by PAR 1 cleavage, the formation of platelet-derived microparticles, and the total thrombocyte count within the blood. These results corroborate the results of the previous in vitro studies that have demonstrated a reduced thrombogenicity after the application of pHPC to small nitinol plates [13]. In our previous study, pHPC-coated and uncoated small nitinol plates were incubated with heparinized human whole blood for 10 min in vitro. Adherent thrombocytes were visualized via a fluorescent CD61^+^ antibody using fluorescence microscopy. A significantly lower number of fluorescent CD61^+^ platelets were seen on pHPC-coated surfaces relative to uncoated nitinol plates (1.12 ± 0.4% vs. 48.61 ± 7.3%; *p* ≤ 0.001). The results of the current study build on our initial in vitro results and demonstrate that the pHPC coating remains effective on the braided wires of the p48 flow diverter under simulated blood flow conditions for 120 min.

The pHPC is designed to replicate the glycocalyx of the arterial wall and is extremely hydrophilic. The mechanism of action of pHPC relates to these properties rather than a pharmacological mechanism of action. Similarly, in vivo studies have shown that the polymer coating does not cause either an acute or a chronic inflammatory reaction nor does it appear to inhibit the neo-endothelialization process [17]. The p48_HPC has recently been granted CE mark and is available for clinical use with SAPT in justified exceptional cases, if no reasonable alternative therapy is given.

The results of this study are promising. However, further work is necessary, as the study has several limitations including the small number of test devices as well as blood donors. Furthermore, there is no aneurysm included in the model and only stent-induced thrombogenicity has been measured. Moreover, the stents were deployed in hollow tubes of uniform radius and uniform curvature that does not accurately represent the intracranial arterial tree and its flow dynamics. For future studies, the comparison of pHPC to other antithrombogenic coatings in the same model might be of interest. Even though the safety of pHPC is proven when implanted in various animal models, the scale of the antithrombogenic effect itself should be further assessed in vivo to gain better understandings of its beneficial potential compared to uncoated devices. Finally, a large-scale clinical study could quantify the antithrombogenic properties of pHPC in humans in clinical cases, especially regarding the question whether the usage of SAPT or no APT at all can be justified for pHPC-coated devices.

## Conclusion

The newly developed phenox Hydrophilic Polymer Coating (pHPC) significantly reduces the thrombogenicity of the p48 flow diverter. This may help reduce the risk of thromboembolic complications when using these devices and minimize the risks associated with dual antiplatelet therapy.
